# Optimization of the Thawing Protocol for Iberian Boar Sperm

**DOI:** 10.3390/ani12192600

**Published:** 2022-09-28

**Authors:** Cristina Tomás-Almenar, Eduardo de Mercado

**Affiliations:** Animal Reproduction Department, National Institute of Agronomic Research (INIA-CSIC), Puerta de Hierro Avenue s/n, 28040 Madrid, Spain

**Keywords:** Iberian boar sperm, freezing, thawing rate, thawing extender

## Abstract

**Simple Summary:**

Limited attention is paid to sperm thawing protocols, and their study could be relevant to endangered species or breeds, especially for cryopreserved material present in the existing gene banks. The aim of this study was to determine the most optimal thawing protocol for Iberian boar sperm through testing different thawing rates and different modifications of the thawing extender. Based on the findings, the most optimal results were obtained thawing at 70 °C for 8 s with the inclusion of cyclodextrins loaded with cholesterol (CLC) in the extender, revealing the importance of adapting the thawing protocols.

**Abstract:**

Thawing protocols have been barely studied, and their modifications may lead to a substantial improvement in post-thawing sperm quality, which could be of great relevance to existing sperm banks, such as those for Iberian pig breeds with varieties in danger of extinction. For that, the study aimed to evaluate different thawing rates and to evaluate modifications in the composition of the thawing extender (basic pH to 8–8.2, incorporation of cyclodextrins loaded with cholesterol [CLC] and the incorporation of ion chelators [EDTA and EGTA]). After thawing, overall sperm motility and kinematic parameters, acrosome status and sperm membrane integrity were evaluated. The most optimal results were obtained with the thawing rate reaching 70 °C for 8 s with the inclusion of 12.5 mg of CLC/500 × 10^6^ spermatozoa in the thawing extender, which showed an improvement compared to the control at 70 °C. In conclusion, to adapt the thawing conditions may be relevant, especially for endangered species or breeds such as some varieties of Iberian pig, since this process could also be used in samples cryopreserved in gene banks.

## 1. Introduction

The Iberian pig is an indigenous breed from Spain which has a high ecological, economic and cultural value, but their number has been drastically reduced due to factors such as African swine fever, the crossbreeding with the Duroc breed or declining interest in the breed by farmers due to greater reproductive problems, especially in the total number of piglets born and the number of piglets born alive [1]. In this context, the creation of gene banks would improve the management of these populations and preserve their genetic value [2]. Cryopreserved sperm is an excellent way to preserve valuable genetic materials [3], but the freezing and thawing protocols negatively affect spermatozoa survival and functionality, which in the last term causes a reduction in reproductive performance [4].

Many studies have shown that it is possible to improve the sperm freezing protocol in porcine species [4,5,6], but the frozen sperm stored in existing animal gene banks may not benefit from such improvements. Therefore, through the improvement of the thawing process, maximum potential in sperm quality could be reached. This process has been barely investigated and plunging the straw in a water bath at 37 °C for 20 s followed by diluting the thawed content 1:1 in Beltsville Thawing Solution (BTS) [6] is the most common protocol to thaw boar sperm.

However, in recent years, it has been observed that some modifications may improve post-thaw sperm quality. These modifications focus on two aspects of the process, the thawing rate and the composition of the thawing extender. Regarding the thawing rate, some authors have shown that rates between 1200 and 1800 °C/min are the most suitable [7,8,9,10], but one aspect to be considered is that the increasing of temperature into the straws is not linear [10,11], and different thawing temperatures with same plunging times lead to different final temperatures in the straw content. Regarding the thawing extender, it should be mentioned that porcine species are practically the only species that use it, with BTS being the most common, although other extenders have been tested. Supplementation of the thawing extender with compounds such as seminal plasma [12,13], cyclodextrins loaded with cholesterol (CLC) [14], antioxidants [15,16,17], ion chelators [18] or the modification of its pH [19] have shown substantial improvements in the sperm membrane integrity and also in the post-thaw movement quality.

Since different resistances against the cryopreservation process have been demonstrated among breeds [20,21,22], as well as the response of Iberian boar sperm to different conservation extenders [23], the aim of this study was to evaluate the most adequate thawing rate for Iberian boar sperm and to test if some improvements made in the thawing extender in crossbreed pigs could be extrapolated to this breed.

## 2. Materials and Methods

### 2.1. Animals, Semen Collection and Freezing-Thawing Protocol

All procedures were conducted in accordance with specific regulations of the Directive of the European Union Council (2010/63/EU) and the Spanish Government [24].

Ejaculates were collected once a week for 6 consecutive weeks from three sexually mature Iberian boars housed in a commercial farm in Spain (ages range from 1.5 to 2 years). The semen samples were obtained using the gloved-hand method and extended in BTS [25] (1:1; *v*/*v*), after which they were transported at 17–18 °C to the laboratory within 2–3 h before experiment. The samples were pooled prior to the centrifugation and cryopreservation. Sperm was evaluated for conventional characteristics using standard laboratory techniques, and all ejaculates presented a concentration ≥200 × 10^6^ sperm/mL, ≥85% of sperm with normal morphology and with ≥75% and ≥80% of motile and viable spermatozoa.

Sperm were cryopreserved using the straw freezing procedure for Iberian boar sperm [26]. Briefly, diluted semen samples were centrifuged at 2400× *g* for 3 min at 15 °C. The resulting pellets were re-suspended in freezing extender (lactose-egg-yolk (LEY); 80% (*v*/*v*) of 310 mM β-lactose and 20% (*v*/*v*) of hen egg yolk with 100 μg/mL kanamycin sulfate as antibiotic; adjusted to 330 ± 5 mOsm/kg and pH 7.2) to a concentration of 1.5 × 10^9^ cells/mL. A programmable thermostatic water bath was used to cool to 5 °C within 120 min, and after that, diluted sperm were re-suspended to a final concentration of 1 × 10^9^ cells/mL with LEY supplemented with glycerol and Equex (LEYGO) extender (92.5% of LEY, 1.5% of Equex STM (Nova Chemical Sales Inc., Scituate, MA, USA) and 6% of glycerol (*v*/*v*); adjusted to 1650 ± 15 mOsm/kg and pH 7.2). The sperm was loaded into 0.5 mL straws (Minitüb, Tiefenbach, Germany), sealed, transferred to a programmable freezer and frozen as follows: from 5 to −5 °C at a rate of 6 °C/min, from −5 to −80 °C at 40 °C/min, held for 30 s at −80 °C, then cooled at 70 °C/min to −150 °C, and plunged into liquid nitrogen (LN_2_). The straws were then stored in LN_2_ until their analysis.

Thawing process was carried out as specified in the experimental design. All samples were incubated in a water bath at 37 °C up to 30 min, the time at which post-thaw sperm quality was assessed.

### 2.2. Experimental Design

#### 2.2.1. Experiment 1: Evaluation of Different Thawing Rates

The thawing rates were obtained by the combination of three different temperatures and different plunge times into the water bath. The thawing rates assayed were compared with a control sample that was thawed with a standard protocol for boar sperm at 37 °C for 20 s [6] (Table 1). To discern between the possible effect of the final reached temperature or thawing rate, three times were selected for each thawing temperature to reach the same final temperature as the control sample (28 °C), with one above (34 °C) and the other below (16 °C).

The combination of temperatures and times were previously tested, and the final temperature reached was determined. For that, the straw content was immediately transferred to a test tube where the temperature was measured by introducing a mono-channel digital record thermometer (Hanna Instruments HI 98509) in the center of the tube.

#### 2.2.2. Experiment 2: Evaluation of Different Thawing Extenders

In this experiment, three modifications of the thawing extender that have shown significant improvements in post-thaw quality in boar sperm were evaluated. The first was the incorporation of cyclodextrins loaded with cholesterol (CLC) at a concentration of 12.5 mg of CLC/500 × 10^6^ spermatozoa [14]. The second was the modification of the pH of the thawing extender to a basic pH of 8–8.2 [19], and the last one was the incorporation of two ion chelators (EDTA and EGTA) at a concentration of 6 mmol/L of EDTA and 6 mmol/L of EGTA [18]. The final concentration of EDTA was adjusted taking into account the concentration of this compound in the BTS.

Each modification was analyzed in samples thawed at 37 °C for 20 s, which was used as control, and also using the thawing rate selected in the previous experiment.

### 2.3. Post-Thaw Sperm Quality Assessments

#### 2.3.1. Assessment of Sperm Motility

A computer-assisted sperm analysis system (Integrated Semen Analysis System, ISAS Proiser, Valencia, Spain) was used to evaluate motility characteristics. The system operated at 25 video frames per second (25 Hz), with settings of particle area from 10 to 80 µm and search radius 11 µm, according to Cremades [27]. When sperm presented an average path velocity (VAP) lower than 10 µm/s, they were classified as non-motile, and when they exhibited a VAP > 45 µm/s and a straightness index (STR) ≥ 45%, they were considered as progressively motile. For that, a pre-warmed (38 °C) Makler counting chamber (Sefi Medical Instruments, Haifa, Israel) with 3 µL aliquot of sperm sample was placed. A minimum of 300 sperm per sample were analyzed (four–five fields), and the following sperm parameters were evaluated: total motile sperm (%TMS) and progressive motility sperm (%PMS). The kinematic values evaluated included the curvilinear velocity (VCL, µm/s), the straightline velocity (VSL, µm/s), the average path velocity (VAP, µm/s), the percentage of linearity (LIN, %), the percentage of straightness (STR, %), the wobble coefficient (WOB, %), the mean amplitude of lateral head displacement (ALH, mm) and finally the means of the beat cross frequency (BCF, Hz).

#### 2.3.2. Assessment of Acrosome Status

**Figure 1:**
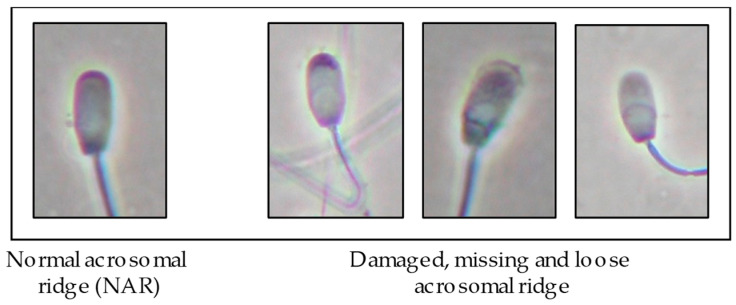
Scoring system to assess of acrosome status. Only the spermatozoa with normal acrosomal ridge (NAR) were considered in the results.

The acrosome status was evaluated by phase contrast microscopy at 1000×. Briefly, samples were fixed in 2% glutaraldehyde and a minimum of 200 acrosomes were examined per sample. The damage to the acrosome cap was classified by the scoring system reported by Pursel [28], and the data are presented in the results as percentage of spermatozoa with normal acrosomal ridge (%NAR; Figure 1).

#### 2.3.3. Assessment of Plasma Membrane Integrity

**Figure 2:**
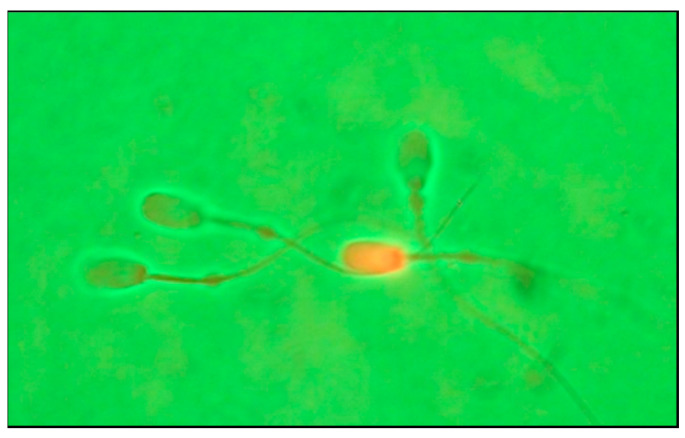
Assessment of plasma membrane integrity. Red stained sperm corresponding to dead sperm and non-red stained corresponding to sperm with intact plasma membrane (SIPM).

Sperm plasma membrane integrity was assessed using phase contrast microscopy (Nikon Eclipse E400, Tokyo, Japan) coupled with a fluorescence system (Nikon C-SHG1) equipped with a mercury lamp (100 W) and a Nikon filter (510/590 nm), according to the technique described by Tomás [29]. For that, sperm samples were stained with 5 µL of propidium iodide (PI, 0.5 mg/mL) and incubated at 37 °C in darkness for 10 min. After placing the sample under phase contrast microscopy, the fluorescence was connected, with non-viable sperm showing red color, and viable sperm were not stained and visible under phase contrast (Figure 2). A minimum of 300 cells per sample in random fields were examined, and the data are presented in the results as percentage of sperm with intact plasma membrane (%SIPM) (non-red stained sperm by PI).

### 2.4. Statistical Analysis

Statistical analyses were performed in the open-source programming R [30] through the Rstudio interface [31]. After testing the normality of the data (Shapiro–Wilk test), a mixed model analysis of variance (ANOVA) was performed to determine the effect of different conditions in the thawing protocol in each experiment. The interaction was included in the model but did not show a significant effect (*p* > 0.05), and thus, the means correspond to each of the effects separately. When ANOVA revealed a significant effect, the means were compared using the Tukey’s multiple comparison test and were considered to be significant at *p* < 0.05. Results are presented as means ± standard deviations. To represent the relation between all variables and the pattern of similarity between observations and variables, a principal component analysis (PCA) was performed. Prior to PCA, all the variables were transformed to the same scale.

## 3. Results

### 3.1. Experiment 1: Evaluation of Different Thawing Conditions

**Figure 3:**
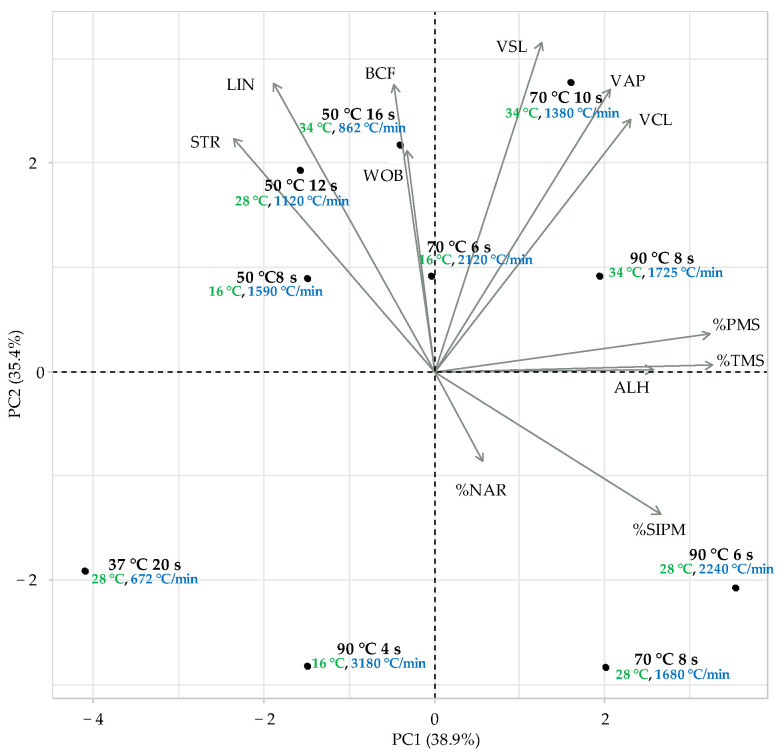
Representation of the first two components of principal component analysis (PCA). %NAR: normal acrosomal ridge, %SIPM: sperm with intact plasma membrane, %TMS: total motile sperm, %PMS: progressive motility sperm, VCL: curvilinear velocity (µm/s), VSL: straightline velocity (µm/s), VAP: average path velocity (µm/s), LIN: linearity (%), STR: straightness (STR, %), ALH: mean amplitude of lateral head displacement (mm), BCF: means of the beat cross frequency (Hz). Green letters indicate the final temperature reached, and blue letters indicate the calculated thawing rate.

The multivariate analysis by PCA was useful to visualize the structure of the data and to identify groups of variables and observations. In Figure 3, the data are represented by the two first components (PC1 and PC2), which explain 74.3% of the total variance. The variables most significant in the PC1 were %TMS, %PMS and %SIPM. Focus on these three variable groups may be identified: the samples thawed at 70 °C for 6 and 8 s and those thawed at 90 °C for 6 and 8 s showed better values, and the samples thawed at 50 °C and at 70 °C for 10 s presented intermediate values, with the samples thawed at 90 °C for 4 s and at 37 °C for 20 s showing worse values. Therefore, it seems more optimal results may be obtained with a thawing rate between 1680 and 2240 °C/min and above or below rates show worse results. It may be noted there was not a clear cluster according to the final temperature reached after thawing (16, 28 or 34 °C).

**Figure 4:**
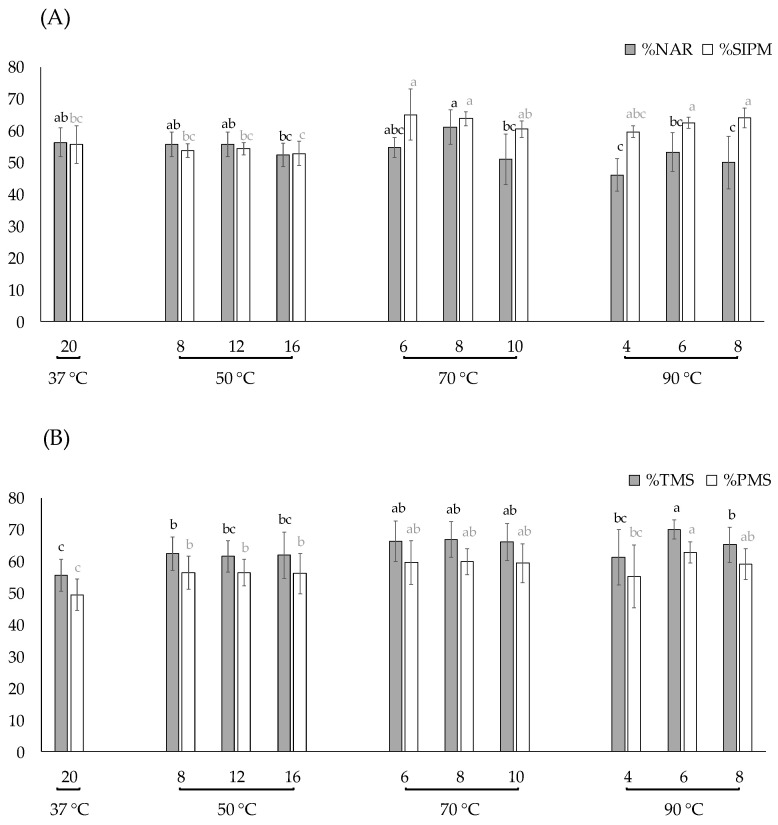
Evaluation of different thawing conditions. Data are expressed as mean ± standard deviation. (**A**) Effect on normal acrosomal ridge (%NAR) and sperm with intact plasma membrane (%SIPM); ^a, b, c^ Indicate significant differences between treatments (*p* < 0.05); black letters are within %NAR and grey letters within %SIPM. (**B**) Effect on total motile sperm (%TMS) and progressive motility sperm (%PMS); ^a, b, c^ Indicate significant differences between treatments (*p* < 0.05); black letters are within %TMS and grey letters within %PMS.

Evaluating the set of all variables through ANOVA, in general high thawing temperatures (70 and 90 °C) showed better values of sperm with %SIPM. This improvement was affected, naturally, by the plunge times into the water bath, obtaining significantly (*p* < 0.05) better results with thawing for 6 and 8 s with respect to the control sample (37 °C for 20 s) and to samples thawed at 50 °C (Figure 4A).

In contrast, the normal acrosomal ridge did not improve with the increasing thawing temperature. The %NAR decreased at 50 and 70 °C when the straws were plunged for longer (16 and 10 s, respectively), and the worst values were with the thawing rates reached with 90 °C, being significantly different (*p* < 0.05) to the control sample for 4 and 8 s.

In reference to sperm motility, for both total motile sperm and progressive motility sperm, the sperm thawed with the standard protocol (37 °C for 20 s) showed the lowest values, being significantly different to the sperm thawed at 70 °C and 90 °C for 6 and 8 s, which showed the best results (Figure 4B). Through its focus on the kinematic parameters, the velocities (VCL, VSL and VAP; Table 2) did not show a clear pattern. In general, most thawing rates tested showed higher velocities than control sample, being significantly different (*p* < 0.05) at 50 °C for 12 and 16 s, at 70 °C for 10 s, and at 90 °C for 8 s. The parameters related to the trajectory as linearity (LIN), straightness (STR) and wobble (WOB) showed barely any differences with the control sample. There were not relevant differences between thawing rates for the beat cross frequency (BCF), while the higher amplitude of lateral head (ALH) displacement were obtained at 50 °C and at 90 °C for 8 s.

Based on the findings of this experiment, the thawing rates obtained with 50 °C were discarded by presenting the worst sperm quality. The samples thawed at 90 °C were also discarded for poor quality in the acrosome status, and the samples thawed at 70 °C showed good overall results; however, finally 70 °C for 8 s was selected for the next experiment for its better quality in the acrosome status.

### 3.2. Experiment 2: Evaluation of Different Modifications in the Thawing Extenders

**Figure 5:**
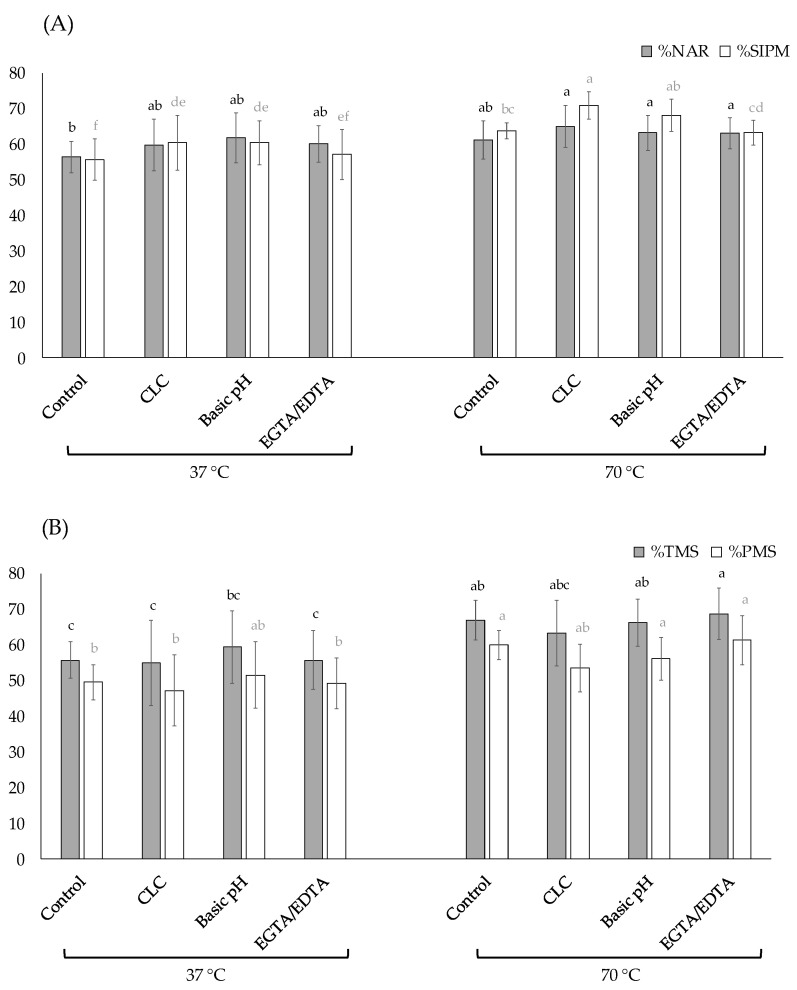
Evaluation of different modifications in the thawing extenders. Data are expressed as mean ± standard deviation. (**A**) Effect on normal acrosomal ridge (%NAR) and sperm with intact plasma membrane (%SIPM). ^a, b, c, d, e, f^ indicate significant differences between treatments (*p* < 0.05); black letters are within %NAR and grey letters within %SIPM. (**B**) Effect on total motile sperm (%TMS) and progressive motility sperm (%PMS). ^a, b, c^ indicate significant differences between treatments (*p* < 0.05), and black letters are within %TMS and grey letters within %PMS.

The three modifications tested in the thawing extender (CLC addition, basic pH or ion chelators addition) revealed significant differences (*p* < 0.05) in the percentage of NAR when the samples were thawed at 70 °C in comparison with the control sample at 37 °C (Figure 5A).

Nevertheless, for the percentage of SIPM the sperm thawed with CLC supplementation at 70 °C showed the highest value, marking a significant difference (*p* < 0.05) with the rest of the protocols, except on sperm thawed in an extender adjusted at basic pH at 70 °C.

In the case of the motility, the sperm thawed at 70 °C, with and without changes in the extender, showed significantly the highest percentage of TMS and PMS with respect to the 37 °C thawing treatments, and only the treatment at 37 °C with basic pH showed significantly similar values to the 70 °C protocols (Figure 5B).

Respecting the individual kinematic parameters (Table 3), the samples thawed at 70 °C with ion chelators (EDTA and EGTA) showed the highest values of velocities being significantly different (*p* < 0.05) to most of the samples thawed at 37 °C, while the samples thawed at 37 °C in modified extenders showed lower values. In reference to the trajectory, the samples thawed at basic pH showed the lowest values in LIN and STR parameters, being significantly different (*p* < 0.05) with respect to the control sample.

For the BCF parameter, samples thawed in the extender supplemented with CLC showed the lowest values and significant different to samples thawed at 37 and 70 °C in the non-supplemented extender. The ALH was higher in the samples thawed at 70 °C than samples thawed at 37 °C, irrespective of the modifications in the extender.

## 4. Discussion

In general, not enough importance is placed on the thawing procedure, and this work shows the relevance of the conditions provided during the process.

Several studies have presented better results of the sperm quality after thawing with the use of high thawing temperatures (70–80 °C) for a relatively short time (8–10 s) [8,9,10,32]. Similar findings have been observed in the present study, obtaining best results with high temperatures (70 and 90 °C). Focusing on the most representative variables (%TMS, %PMS and %SIPM; Figure 3 and Figure 4) the most optimal thawing rates were reached at 70 °C for 6 or 8 s and 90 °C for 6 or 8 s, in other words, maintaining velocities between 1680 and 2240 °C/min. The slowest rate (37 °C 20 s; 672 °C/min) and the fastest rate (90 °C 4 s; 3180 °C/min) presented the poorest quality, while thawing rates between 862 and 1590 °C/min showed intermediate values.

When the straw is plunged into the water bath, the rise of the internal temperature is not linear [10,11,33]. These authors observed that the yield curve was less pronounced in the range between 0 and 37 °C, being also influenced by the geometry of the straw and even by the composition of the freezing extender.

High thawing temperatures allow a fast pass through the re-crystallization phase (−50 to 0 °C) [10,34,35], which is a danger zone with ice crystal formation causing sperm to switch directly from the glassy state to the liquid state [36] and thereby preventing cellular damage. In addition, there is also a fast pass through the ‘warm shock’ [37] temperatures (0 to 15 °C), which is the inverse process to ‘cold shock’ [38] during rewarming.

This explains why in the present study, the %SIPM improved when the thawing temperature was high (70–90 °C) irrespective of the final temperature reached. A similar tendency was observed for the total and progressive sperm motility. In contrast, the status of acrosome was affected by the highest temperature (90 °C), and this is also showed for other authors in thawing boar sperm [7,37]. This damage in acrosomes could be influenced by the presence of glycerol from the freezing extender [39]. Slight decrease in the content of glycerol (4% vs. 6%) showed a significant enhancement of the post-thawing acrosome status and was not observed as an effect on the acrosome using high thawing temperatures in samples frozen with this extender low in glycerol [40]. Similarly, Athurupana [10] did not observe a detrimental effect on the acrosomes with a thawing at 80 °C without presence of glycerol. This is in line with our results, where the sperm thawing at 90 °C showed good results for membrane integrity and motility but with poorer results in acrosome status.

Several studies observed that an increase in the thawing temperature improved the post-thawed motility [8,9,10,32,35,41,42,43], but a limited number of them evaluated the quality of the movement through the kinematic parameters. In general, they observed that an increase in the thawing temperature (60–70 °C) not only improved the total and progressive motility but also increased the velocities (VCL, VSL and VAP) [9,42,43], similar trends as those observed in our results.

In reference to intermediate thawing temperature (50 °C), this did not show better results than the control sample (37 °C, 20 s), maybe because the rate obtained with this temperature avoids an adequate reduction of the size of the crystals during the recrystallization, causing severe damage to sperm [44]. The movement pattern observed in the samples thawed at 50 °C was marked by high values of velocities and ALH, parameters related to the sperm hyperactivation [45], which might be linked to a lower thawing velocity when pass through ‘warm shock’ phase, which might imply a higher calcium influx into the sperm cell and consequent hyperactivation [46].

Based on the findings, the thawing conditions of 70 °C for 8 s, corresponding to 1680 °C/min, were considered the most optimal for Iberian boar sperm. Similar conditions were observed by other authors in other pig breeds [8,9,10], as well as in other species [35,42].

The thawing extenders have been barely studied, but light modifications may substantially improve post-thawing sperm quality [12,13,14,15,16,17,18,19,47].

In the present study, three changes in the thawing extender were selected according to the results obtained in previous works. The addition of CLCs in the thawing extender has shown a significant improvement in the sperm membrane integrity at 37 °C [14], with similar effects observed at 37 °C, and at 70 °C the results were even better. This effect in the membrane could be due to CLCs improving the interaction between lipids, decreasing the membrane permeability and increasing its stability [48].

On the other hand, the change of pH to basic in thawing extender may improve the post-thawing sperm motility [19]. In this case, we did not observe a significant improvement with the use of basic pH at 37 °C nor at 70 °C. During the cryopreservation process, it has been observed that the boar sperm has its capability to release calcium altered or modified. The calcium is accumulated into the cell affecting the membrane potential [49], thus causing a decrease in the sperm motility. An increase in the internal pH [50,51] would induce a hyperpolarization of the membrane, and consequently an activation of the calcium channels [52,53]. Since the internal pH can be modified through changes in the external pH [54], basic pH in the thawing extender could increase the motility in those spermatozoa with poor motility or non-motile but with intact membrane integrity. In contrast to our previous study [19], in this case there is barely any difference between %SIPM and %TMS; therefore, there is no room for improvement for motility.

In the last term, the addition of ion chelators showed an improvement in the post-thawing sperm quality, especially in the motility [18]. These researchers demonstrated that the inclusion of EDTA and EGTA in the thawing extender implied a reduction in the calcium flux into the cell. Consequently, the effects derived from the calcium accumulation would be avoided [49] since high intracellular calcium levels inhibit sperm motility [55]. Similarly to basic pH, there was not a significant enhancement in the quality parameters analyzed at 37 °C or at 70 °C, although it is worth mentioning that this treatment thawing at 70 °C showed the greater values for total and progressive motility.

Regarding kinematic parameters, similar to the results observed in the first experiment, the samples thawed at 70 °C showed higher velocities (VCL, VSL and VAP) and ALH, the highest values corresponding to thawing at 70 °C with the addition of ion chelators (EDTA and EGTA). The CLCs provoked a significant decrease in BCF parameter at 37 and 70 °C, which could be related to the greater cholesterol content in the membrane that consequently reduced the cryocapacitation phenomenon [56]. The basic pH treatment for both thawing temperatures (37 and 70 °C) showed a significant decrease in relation to their respective control samples in VSL, LIN and STR, presenting a more circular movement pattern. Calcium levels can modify the trajectory and the type of movement [46,57], and for this reason the basic pH, modifying the activation of calcium channels, which might have influenced the modification of this movement pattern.

## 5. Conclusions

Based on the findings, it may be concluded that the best thawing conditions for Iberian boar sperm was a thawing rate of 1680 °C/min (70 °C for 8 s), and with the inclusion of 12.5 mg of CLC/500 × 10^6^ spermatozoa in the thawing extender, the membrane integrity could be improved. Other modifications in the thawing extender, such as basic pH or the addition of ion chelators (EDTA and EGTA) could increase the sperm motility, but further studies are required to determine the underlying mechanism of these changes. Thus, adapting the thawing conditions might be relevant, especially in endangered species or breeds such as some varieties of Iberian pig, since this could also be used in existing samples cryopreserved in gene banks.

## Figures and Tables

**Table 1:** Thawing conditions evaluated.

Thawing Temp. (°C)	Plunge Time (s)	Final Temp. (°C)	Thawing Rate (°C/min)
37 (control)	20	28	672
50	8	16	1590
	12	28	1120
	16	34	862
70	6	16	2120
	8	28	1680
	10	34	1380
90	4	16	3180
	6	28	2240
	8	34	1725

**Table 2:** Evaluation of different thawing conditions: Effect on kinematic parameters.

Thawing Temp. (°C)	Plunge Time (s)	Final Temp. (°C)	Rate (°C/min)	VCL	VSL	VAP	LIN	STR	WOB	ALH	BCF
37	20	28	672	73.5 ± 4.5 ^c^	53.3 ± 4 ^d^	63.6 ± 4 ^b^	72.5 ± 3.9 ^b^	83.8 ± 3.5	86.5 ± 1.3 ^ab^	2.28 ± 0.1 ^de^	8.1 ± 0.6 ^bcd^
50	8	16	1590	79.5 ± 8.3 ^abc^	58 ± 6.4 ^abcd^	69.1 ± 8.6 ^ab^	73 ± 4 ^ab^	84.1 ± 3.3	86.7 ± 3.2 ^a^	2.58 ± 0.16 ^a^	8.05 ± 0.1 ^cd^
	12	28	1120	80.6 ± 5.2 ^ab^	59.4 ± 3.4 ^abc^	70.7 ± 4.5 ^a^	74.2 ± 4.3 ^a^	84.4 ± 3.4	87.9 ± 1.8 ^a^	2.54 ± 0.21 ^ab^	8 ± 0.2 ^d^
	16	34	862	84.1 ± 8.1 ^a^	61 ± 5.9 ^ab^	72.2 ± 7.7 ^a^	72.6 ± 5.1 ^ab^	84.7 ± 4.4	85.7 ± 2.7 ^ab^	2.45 ± 0.21 ^abc^	7.9 ± 0.3 ^d^
70	6	16	2120	79.3 ± 7.9 ^abc^	57.7 ± 3.5 ^bcd^	69.2 ± 7 ^ab^	73.1 ± 4.7 ^ab^	83.7 ± 5.4	87.2 ± 0.5 ^a^	2.4 ± 0.19 ^bcd^	8.7 ± 0.4 ^a^
	8	28	1680	79.7 ± 9.5 ^abc^	55.2 ± 4.6 ^cd^	67.8 ± 8.1 ^ab^	69.6 ± 4.5 ^ab^	81.7 ± 3.9	85.1 ± 2.3 ^ab^	2.32 ± 0.25 ^cde^	8.2 ± 0.2 ^bcd^
	10	34	1380	86.2 ± 5.2 ^a^	62.7 ± 3.2 ^a^	74.5 ± 3.7 ^a^	72.9 ± 3.5 ^ab^	84.2 ± 2.8	86.5 ± 1.5 ^ab^	2.23 ± 0.23 ^e^	8.5 ± 0.4 ^ab^
90	4	16	3180	75.5 ± 3.7 ^bc^	53.6 ± 5.5 ^d^	63.9 ± 4.5 ^b^	70.8 ± 5.1 ^b^	83.8 ± 4.1	84.5 ± 2.8 ^b^	2.33 ± 0.14 ^cde^	8.4 ± 0.3 ^abc^
	6	28	2240	81.1 ± 7.5 ^ab^	56.5 ± 4.5 ^bcd^	70.2 ± 6.8 ^ab^	69.7 ± 3.1 ^b^	80.7 ± 2.3	86.7 ± 2.1 ^a^	2.31 ± 0.21 ^cde^	8.2 ± 0.1 ^bcd^
	8	34	1725	84.4 ± 4.5 ^a^	60.7 ± 2.4 ^ab^	73.2 ± 2.6 ^a^	72 ± 4.1 ^ab^	83 ± 3.9	86.4 ± 1.7 ^ab^	2.51 ± 0.2 ^ab^	8.5 ± 0.3 ^ab^

VCL: curvilinear velocity (µm/s), VSL: straightline velocity (µm/s), VAP: average path velocity (µm/s), LIN: linearity (%), STR: straightness (%), WOB: wobble (%), ALH: mean amplitude of lateral head displacement (mm), BCF: means of the beat cross frequency (Hz). Data are expressed as mean ± standard deviation. ^a, b, c, d, e^ indicate significant differences between treatments (*p* < 0.05).

**Table 3:** Evaluation of different modifications in the thawing extenders: effect on kinematic parameters.

Thawing Temp. (°C)	Modifications	VCL	VSL	VAP	LIN	STR	WOB	ALH	BCF
37	Control	73.5 ± 4.5 ^bcd^	53.3 ± 4 ^ab^	63.6 ± 4 ^abc^	72.5 ± 3.9 ^a^	83.8 ± 3.5 ^ab^	86.5 ± 1.3 ^a^	2.28 ± 0.1 ^cd^	8.1 ± 0.6 ^ab^
	CLC	67.5 ± 6.1 ^d^	48 ± 6.4 ^bc^	57.9 ± 6 ^c^	71.1 ± 7.5 ^ab^	82.8 ± 5 ^abc^	85.7 ± 4.3 ^ab^	2.22 ± 0.25 ^cd^	7.35 ± 0.8 ^cd^
	Basic pH	67.9 ± 2.3 ^d^	45.5 ± 4.2 ^c^	57.6 ± 3.4 ^c^	67 ± 5.9 ^bc^	78.9 ± 5.3 ^cd^	84.8 ± 3.2 ^ab^	2.18 ± 0.13 ^d^	8.3 ± 0.4 ^a^
	EGTA/EDTA	72.1 ± 5.9 ^cd^	52.2 ± 5.3 ^ab^	61.4 ± 5.9 ^bc^	72.4 ± 3.7 ^a^	85.2 ± 3 ^a^	85 ± 1.9 ^ab^	2.35 ± 0.12 ^bcd^	7.9 ± 0.6 ^ab^
70	Control	79.7 ± 9.5 ^ab^	55.2 ± 4.6 ^a^	67.8 ± 8.1 ^ab^	69.6 ± 4.5 ^ab^	81.7 ± 3.9 ^abc^	85.1 ± 2.3 ^ab^	2.32 ± 0.25 ^ab^	8.2 ± 0.2 ^ab^
	CLC	72.8 ± 7.1 ^bcd^	49.4 ± 4.6 ^bc^	61.2 ± 5.3 ^bc^	68.1 ± 6.5 ^abc^	80.7 ± 4.8 ^bc^	84.2 ± 3.2 ^ab^	2.4 ± 0.29 ^bcd^	7.1 ± 0.4 ^d^
	Basic pH	75.9 ± 4.8 ^abc^	48.5 ± 3.6 ^bc^	63.9 ± 4.8 ^abc^	63.9 ± 2.3 ^c^	75.8 ± 2.6 ^d^	84.2 ± 1.7 ^ab^	2.43 ± 0.13 ^abc^	8.2 ± 0.3 ^ab^
	EGTA/EDTA	82.5 ± 8.3 ^a^	68.8 ± 7.2 ^a^	68.5 ± 7.7 ^a^	68.5 ± 4.5 ^abc^	82.2 ± 3.4 ^abc^	83.3 ± 2.5 ^b^	2.65 ± 0.18 ^a^	7.8 ± 0.7 ^bc^

VCL: curvilinear velocity (µm/s), VSL: straightline velocity (µm/s), VAP: average path velocity (µm/s), LIN: linearity (%), STR: straightness (STR, %), WOB: wobble (%), ALH: mean amplitude of lateral head displacement (mm), BCF: means of the beat cross frequency (Hz). Data are expressed as mean ± standard deviation. ^a, b, c, d^ indicate significant differences between treatments (*p* < 0.05).

## Data Availability

Not applicable.
